# Estimation of French cattle herd immunity against bluetongue serotype 8 at the time of its re-emergence in 2015

**DOI:** 10.1186/s12917-018-1388-1

**Published:** 2018-03-02

**Authors:** L. Bournez, L. Cavalerie, C. Sailleau, E. Bréard, G. Zanella, R. Servan de Almeida, A. Pedarrieu, E. Garin, I. Tourette, F. Dion, P. Hendrikx, D. Calavas

**Affiliations:** 1 ANSES (French Agency for Food, Environmental and Occupational Health & Safety), Unité de coordination et d’appui à la surveillance, Direction des laboratoires, Maisons Alfort, France; 2 Ministère en charge de l’Agriculture, Direction générale de l’Alimentation, Bureau de la santé animale, Paris, France; 3 ANSES, Laboratoire de santé animale, Université Paris-Est, Maisons-Alfort, France; 4 CIRAD, UMR ASTRE, Montpellier ; Inra, UMR ASTRE, Montpellier, France; 5 Coop de France, Paris, France; 6 GDS France, Paris, France; 7 Races de France, Paris, France; 8 ANSES, Laboratoire de Lyon, Unité Epidémiologie, Laboratoire de Lyon, Lyon, France

**Keywords:** Bluetongue, Serotype 8, Seroprevalence, Herd immunity, Re-emergence, France, Cattle

## Abstract

**Background:**

From 2006 to 2010, France experienced two bluetongue epidemics caused by serotype 1 (BTV-1) and 8 (BTV-8) which were controlled by mass vaccination campaigns. After five years without any detected cases, a sick ram was confirmed in August 2015 to be infected by a BTV-8 strain almost identical to that circulating during the previous outbreak. By then, part of the French cattle population was expected to be still protected, since bluetongue antibodies are known to last for many years after natural infection or vaccination. The objective of this study was to estimate the proportion of cattle in France still immune to BTV-8 at the time of its re-emergence in 2015.

**Results:**

We used BTV group-specific cELISA results from 8525 cattle born before the vaccination ban in 2013 and 15,799 cattle born after the ban. Samples were collected from January to April 2016 to estimate seroprevalence per birth cohort. The overall seroprevalence in cattle at national and local levels was extrapolated from seroprevalence results per birth cohort and their respective proportion at each level. To indirectly assess pre-immune status of birth cohorts, we computed prevalence per birth cohort on infected farms in autumn 2015 using 1377 RT-PCR results. These revealed limited BTV circulation in 2015. Seroprevalence per birth cohort was likely to be connected to past exposure to natural infection and/or vaccination with higher seroprevalence levels in older animals. A seroprevalence of 95% was observed for animals born before 2008, of which > 90% were exposed to two compulsory vaccination campaigns in 2008-2010. None of the animals born before 2008 were found to be infected, unlike 19% of the young cattle which had never been vaccinated. This suggests that most ELISA-positive animals were pre-immune to BTV-8. We estimated that 18% (from 12% to 32% per *département*) of the French cattle population was probably pre-immune in 2015.

**Conclusions:**

These results strongly suggest a persistence of antibodies for at least 5-6 years after natural infection or vaccination. The herd immunity of the French cattle population probably limited BTV circulation up to 2015, by which time more than 80% of cattle were naive.

**Electronic supplementary material:**

The online version of this article (10.1186/s12917-018-1388-1) contains supplementary material, which is available to authorized users.

## Background

Bluetongue (BT) is a vector-borne viral disease of wild and domestic ruminants that can cause major losses in ruminant production especially in sheep. The BT virus (BTV) is transmitted by several species of biting midges of the genus *Culicoides*. To date, 27 BTV-serotypes have been identified [1]. In 2006, the emergence of BTV serotype 8 (BTV-8) in northern Europe initiated a widespread epidemic from 2006 to 2009 in central and western Europe [2]. In France, BTV-8 was introduced by the end of 2006 from Belgium and spread over most of the country, infecting at least 42,850 farms between 2006 and 2009 [3]. During the same period, France experienced another BT epidemic with BTV serotype 1 (BTV-1), first detected in November 2007 close to the Spanish border. BTV-1 mainly circulated in south-western France, although a few infected (i.e. RT-PCR positive) animals were later discovered throughout France, without clear evidence of any local BTV circulation. Two years of mandatory vaccination followed by two voluntary campaigns were launched against both serotypes in throughout the French mainland from November 2008 to April 2010, and from November 2010 to April 2012 respectively. The cattle vaccination coverage against both serotypes was estimated to be 80% in 2008-2009, 90% in 2009-2010 and 25-30% in 2010-2011 [3, 4]. The vaccination coverage is unknown for 2011-2012 but assumed to be lower than in 2010-2011 due to both lack of interest and reluctance by farmers. The number of outbreaks drastically decreased in 2009 probably due to the high proportion of naturally infected (and thus immunised) and vaccinated animals. The last BTV-8 and BTV-1 outbreaks were detected respectively in December 2009 and June 2010, and mainland France officially recovered a BT-free status in December 2012. Vaccination was banned in mainland France on 31th May 2013.

On 11th September 2015, French authorities notified to the OIE a BTV-8 outbreak in the Allier *département* in central France. The virus was detected in a 5-year-old ram which showed clinical signs evocative of BT. The virus’s genetic sequence was 99.9% similar to the virus circulating in 2006-2009 [5]. BTV-8 eradication through mandatory vaccination was not carried out in France in 2015. The limited amount of vaccines available in 2015-2016 was mainly used for animals leaving the restriction zone (i.e. within 150 km of outbreaks). From August 2015 to June 2016, 284 outbreaks were detected through pre-export tests (74%), active surveillance (21%) and clinical surveillance (5%), and were mainly located in the centre of France (the Allier and Puy-de-Dôme *départements*).

One of the main hypotheses for the resurgence of BTV-8 in France is that the virus had been circulating at low levels since 2009 and had remained undetected by the surveillance system [6–8]. Like other viral diseases, the intensity of BTV circulation may have increased along with the increasing proportion of naive animals due to ruminant population turn-over and/or loss of immunity. One can expect resurgence to occur when the proportion of naive animals towards BTV-8 is sufficiently high to lead to more intensive viral circulation and spread, but the value of this threshold is unknown. Cattle are much more attractive to *Culicoïdes* spp. than are sheep [9, 10] and hence more frequently infected by BT viruses [11]. They are considered as the primary reservoir and amplifying host for the virus [12, 13]. In France, they are much more numerous than sheep, with 19.2 and 7 million of head respectively (source: the French Livestock Institute Idele and GEB). By July 2015, 23% of the French cattle population was composed of animals born before 2010, which had therefore been present during the mandatory vaccination campaigns of 2008-2010 (source: National Identification Database BDNI). Some of these animals might still be immune to BTV-8, but their proportion was unknown. Such data is not available for sheep.

The duration of BTV-8 immunity acquired after natural infection or vaccination and how it decreases over time depends on several factors. The protective immune status of animals with respect to BTV is generally assessed via their humoral immune response, even though cellular immune response might also be a determinant [14–17]. Although neutralising type-specific antibodies are generally preferred for estimating BT protective immune status, group-specific antibodies detected by ELISA can also be used to infer the immune status of animals against a serotype if this one serotype has been circulating or was targeted for vaccination in the area. Seroneutralising and ELISA results are relatively well correlated, although the proportion of ELISA positive results is generally higher [18–21]. Neutralising and group-specific antibodies against BTV-8 have been detected in cattle four years after natural infection and vaccination [18, 21–23]. However, different studies have observed large variation in the proportion of seropositive animals one year after vaccination ranging from 60% to 97% when evaluated by ELISA [18–20, 24–28]. Such a variation could be explained by a difference in the vaccination protocol (e.g. with or without a booster vaccination), the type of vaccine itself or the mean age of animals at vaccination [27]. In France, several inactivated commercial vaccines against BTV-8 and BTV-1 were successively used from 2008 to 2012 (Table 1). According to their birth date, animals received from one to several doses against serotype 1 or 8 (Table 2). All these factors may have influenced the proportion of cattle still immune in France in 2015, and made it difficult to infer this proportion without further investigation.

**Table 1:** Vaccine products used from 2008 to 2013 in France against BTV-8 and BTV-1 in cattle

Year	BTV-8 vaccine	BTV-1 vaccine
2008-2009 (mandatory vaccination campaign)	Bovilis BTV-8® (Intervet, The Netherlands)	Zulvac® 1 Bovis (Fort Dodge Animal health, The Netherlands),Bluevac® 1 (CZ Veterinaria S.A., Spain)
2009-2010 (mandatory vaccination campaign)	BTVPUR® AlSap 8 (Merial, France)	Zulvac® 1 Bovis (Fort Dodge Animal health, The Netherlands), Bluevac® 1 (CZ Veterinaria S.A., Spain)
2010-2013 (voluntary vaccination campaign)	BTVPUR® AlSap 8, Bovilis BTV-8®, Zulvac® 8 Bovis (Fort Dodge Animal health, The Netherlands)	BTVPUR® AlSap 1, Bluevac® 1, Zulvac® 1 Bovis

**Table 2:** Exposure to BTV-8 and vaccination campaign characteristics per cattle birth cohort in France

Birth cohort	BTV-8 exposure	Vaccination campaign (estimated vaccination rate (%))			
	2007-2009	Mandatory 2008-2009 (~ 80%)	Mandatory 2009-2010 (~ 90%)	Voluntary 2010-2011 (~ 20-30%)	Voluntary 2010-2011 (unknown)
< July 2008	Yes	Yes	Yes	Yes	Yes
July 2008 - June 2009	Yes	Calves*	Yes	Yes	Yes
July 2009 - June 2010	No	No	Calves*	Yes	Yes
July 2010 - June 2011	No	No	No	Calves*	Yes
July 2011 - June 2012	No	No	No	No	Calves*
July 2012 - June 2013	No	No	No	No	No
> June 2013	No	No	No	No	No

*Cattle up to 12 months-old

The objective of this paper was to estimate the proportion of cattle still immune to BTV-8 in France in 2015 at national and local levels. This was essential to better understand BT epidemiology and to empirically estimate the threshold levels of the proportion of naive animals in the cattle population required for BTV to re-emerge and spread at a detectable level. This was also important to better tailor surveillance measures and to provide advice on vaccination to French farmers. Considering the expected differences in exposure to viral infection over time and the successive vaccination protocols (Table 2), we estimated the proportion of seropositive cattle per birth cohort (see definition below) in winter 2015-2016 using a BTV group-specific competitive ELISA of 15,799 cattle and extrapolated it to the cattle population as a whole. In order to check the hypothesis that infection prevalence in autumn 2015 was lower in birth cohorts which were the more exposed to BTV-8 infection or vaccination, we estimated the proportion of cattle positive to BTV-8 by RT-PCR per birth cohort on infected farms in autumn 2015. According to this hypothesis, we expected to find a lower number of infected animals in older birth cohorts.

## Methods

### Estimates of seroprevalence in cattle per birth cohort and the *département*’s BTV-8 status in 2015

#### Definition of birth cohorts and status of BTV-8 infected/non-infected areas

Given the differences in cattle exposure to BTV-8 and the different vaccination protocols implemented from 2007 to 2016, seroprevalence was expected to vary between animal birth dates (Table 2). We defined annual birth cohorts from 1st July to 30th June of the following year in order to take into account the period of BTV circulation (mainly from July to November, with a detection window by RT-PCR up to six months post-infection in cattle, i.e. up to May-June) and of vaccination campaigns (conducted during annual surveillance sampling for brucellosis and infectious bovine rhinotracheitis from October to April).

In order to estimate seroprevalence levels before and after the 2015 resurgence, seroprevalence per birth cohort was estimated separately for areas “infected” and “non-infected” by BTV-8 in 2015-2016. To define infected and non-infected areas, we chose the “*département”,* the French administrative unit that is also the geographical area for BT management. A *département* was considered “infected” in 2015 when at least one animal was found RT-PCR-positive by the surveillance system between 1st August 2015 and 30th June 2016 [29]. It is worth noting that a very high number of cattle from France (> 140,000) were tested by RT-PCR mainly for pre-export tests during this period. Blood samples were tested by a BTV group-specific RT-PCR and positive samples were then screened with a BTV-8 type-specific RT-PCR. These analyses were carried out by local veterinary laboratories. Results with a Ct value between 35 and 39 were confirmed by the National Reference Laboratory (ANSES, Maisons-Alfort). Given the long persistence of BT antibodies post-infection detected by ELISA, and the detection of viral genome up to six months post-infection by RT-PCR [30, 31], we used RT-PCR-positive results as a proof of an infection occurring in 2015-2016. The date of 30th June was defined as the end of the estimated period of the detection of the virus circulation in 2015-2016 considering several criteria: no vector activity between January to April-May [32], the possible detection of viral genome by RT-PCR up to 6 months post-infection [30, 31], an increase in the number of outbreaks associated with a decrease in Ct values of RT-PCR-positive results in July compared to previous months and first virus isolation in August 2016 [29].

**Fig. 1:**
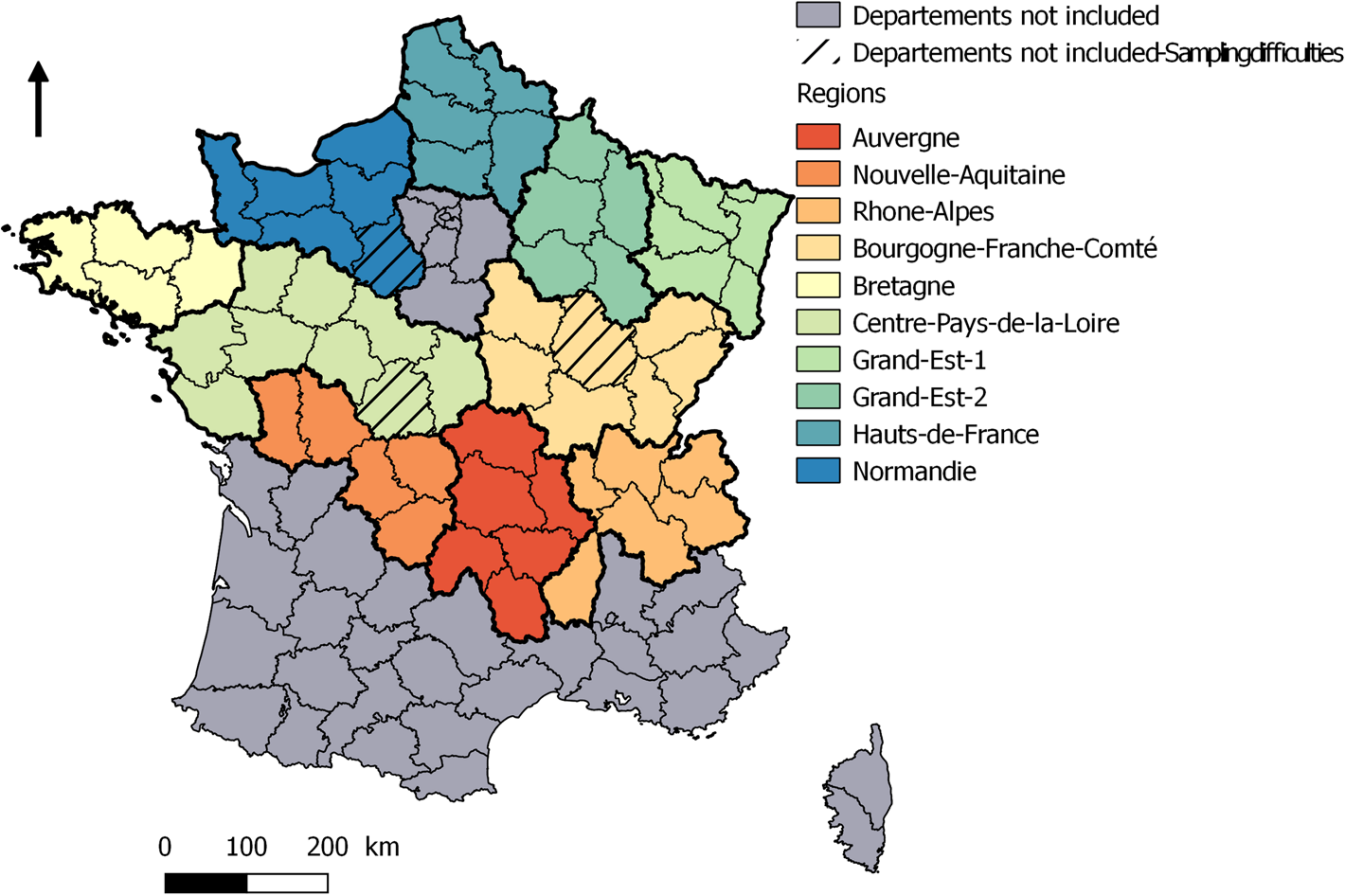
Study area of the 2015 BTV seroprevalence survey in France. In colour, the *départements* (thin black lines) and regions (thick black lines) included in the survey. In grey, *départements* excluded from the survey because of the low number of cattle or BTV-1 circulation in 2007-2008 to remove the effects of past BTV-1 circulation on the antibody response of cattle to BTV-8

*Départements* located in the main BTV-1 infected area in 2007-2008 (South-West France) were excluded to remove potential effects of past BTV-1 circulation on the antibody response of cattle to BTV-8, although a cross-immunity between these two serotypes is not expected [33]. In other *départements* we considered that BTV-1 circulation in 2007-2010 was too small to have a big influence on the response of BTV-8 antibodies. *Départements* with less than 20,000 cattle were also excluded (Fig. 1).

#### Sampling design

For cattle born after January 2013, we used data collected from the national serological survey organised by the French Ministry of Agriculture and carried out from December 2015 to March 2016 (see technical instruction DGAL/SDSPA/2016-35). Its objectives were to detect the presence of BTV in uninfected *départements* and to demonstrate *seasonally-free zones* of BT within the restriction zone. Animals born after January 2013 were targeted as they were considered to be naive and not vaccinated against BT; calves of less than 12 months old were excluded due to the potential persistence of colostral antibodies [34]. Between 167 and 680 cattle from 11 to 47 farms were sampled per *département,* according to the sampling scheme (Additional file 1).

For cattle born before January 2013, sampling was organised at the same time as the national survey of young animals in the winter of 2015-2016. The sampling scheme was adapted to practical and financial constraints inherent to surveillance programmes, especially in the context of re-emergence when animal health officers in the field are regularly asked to conduct surveys/investigations. More specifically, it was designed to limit the amount of work required of them and avoid delaying BT investigation in young animals. Therefore, the sampling scheme was not stratified per birth cohort. Ten farms were randomly selected per *département* and 15 animals not vaccinated in 2015 were randomly sampled per farm in order to reach 150 animals per *département*. This number was considered sufficient to have good estimates of the seroprevalence per birth cohort and per BTV-8 *département* status in 2015.

#### Serological analyses

BTV antibodies were detected in cattle serum by certified local veterinary laboratories using one of the authorised competitive ELISAs. Of the 37 laboratories involved in the surveillance system, 33 used ID Screen Bluetongue Competition (ID VET, France) and four used IDEXX Bluetongue Competition Ab (IDEXX, United Kingdom) (4% of the analyses). Analyses were performed and interpreted according to the manufacturers’ instructions. For the ID-VET kit, samples with a competition percentage ≤ 35%,]35 -45%], > 45% were considered positive, doubtful or negative in that order. For the IDEXX kit, samples with a competition percentage ≤ 70%,]70 - 80%], and > 80% were considered positive, doubtful or negative in that order.

#### Seroprevalence estimates per birth cohort at national and regional levels

Animals that had moved from one *département* to another after July 2015 — considered the potential beginning of BTV circulation in 2015-2016 — were excluded from the analysis.

We calculated seroprevalence per birth cohort and *département* status (infected vs. non-infected in 2015-2016 as defined above) at national level. Confidence intervals of 95% (95% CI) were calculated using exact binomial law. Seroprevalences values of birth cohorts and *départment* status were compared with the Chi^2^ test.

In order to assess if seroprevalence per birth cohort was spatially heterogeneous in 2015, we calculated seroprevalence and its 95% CI per birth cohort and region (an administrative unit including several *départements*; see Fig. 1). This analysis was carried out per region in order to reach a sufficient number of animals per birth cohort to enable birth period seroprevalence estimates within +/− 10%. The median and the range proportion of seroprevalence per birth cohort and per region, and the median range of the seroprevalence’s 95% CI were then calculated.

### Estimates of the seroprevalence of cattle population in France in 2015 at national, regional and *département* levels

As the distribution of birth cohorts is spatially hetergenous in France, we estimated the overall seroprevalence at three geographical levels: national, regional and *département* levels. To do so, we combined the estimate of the seroprevalence per birth cohort and the proportion of animals from each birth cohort in the cattle population of July 2015 at the defined geographical level.

We estimated the national seroprevalence in the cattle population given the estimate of seroprevalence per birth cohort as calculated above. We estimated this seroprevalence per *département* status: “BTV-infected in 2015” or “BTV-non-infected in 2015”. We used the lower and upper limits of the 95% CI of the seroprevalence per each birth cohort to estimate the lower and upper bounds of the confidence interval for cattle seroprevalence. No serological data were available for calves less than 1 year old, but colostral antibodies are present only a few months after birth. We therefore considered them as naïve with a seroprevalence of 0%.

We estimated seroprevalence per region using the seroprevalence levels per birth cohort observed per region. The objective was to assess the spatial variation of seroprevalence due to the spatial variation of the cattle population and of the seroprevalence levels per birth cohort.

Finally, to estimate the seroprevalence of BT in cattle at *département* level, we used the national seroprevalence levels for each birth cohort estimated for the area considered free of BTV infection in order to exclude any effects of the 2015 BTV circulation.

### Proportion of infected cattle per birth period on infected farms in autumn 2015 (RT-PCR)

In order to roughly estimate the likelihood of an animal being infected in 2015 according to its birth cohort, we calculated the proportion of RT-PCR positive animals per birth cohort on infected farms in autumn 2015. We used the results of two other surveys conducted in autumn 2015, organised by the Ministry of Agriculture.

We used RT-PCR results from 609 cattle on ten farms found to be infected during a survey conducted in September 2015. This survey targeted farms located within 2 km of the first BTV-8 outbreak detected in August 2015 in the Allier *département*. We also used results from 768 cattle on 26 farms found to be infected during a national cross-sectional survey conducted in September-October 2015 testing 30 cattle per farm on 1338 farms. All these infected farms were located in central France and were separated by a maximum distance of 220 km. Therefore, we considered that these animals were almost identically exposed to virus circulation in 2015 and were aggregated for the analysis of prevalence per birth period.

Data on birth date and cattle movements were extracted from the National Identification Database (BDNI). All analyses were performed using R software version 3.1.2 [35]. The protocol was designed, and the results analysed and interprated within the “BT working group” of the French Platform for Epidemiological Surveillance in Animal Health (“ESA Platform”).

## Results

### Estimates of seroprevalence in cattle per birth period and the *département*’s BTV-8 status in 2015

A total of 8525 animals born before 2013 and 15,799 animals born after 2013 from 55 *départements* were tested by ELISA. Because of difficulties related to sample collection, laboratory analyses or data recording, three *départements* were excluded from the analysis (Fig. 1). Among the sampled animals, 681 (2.8%) had left their *département* of birth since July 2015 and were therefore excluded from the analysis.

Nationally, we found large seroprevalence differences according to birth periods (Chi^2^ = 16,950, df = 6, *p* < 0.001), with higher levels among the birth cohorts before June 2009 (Table 3). No significant difference in seroprevalence was found between infected and non-infected *départements* in 2015 among animals born before July 2010 (Table 3). A difference of 2% to 6% was observed between infected and non-infected *départements* for animals born after July 2010. Similarly, a seroprevalence of 2-3% was also observed in animals born after July 2013 (animals that had never been vaccinated) and between July 2012 and June 2013 (animals that had probably never been vaccinated) in infected *département*s*,* while it was very low (0.5%) in non-infected *département*s.

**Table 3:** BTV-8 seroprevalence in French cattle in the winter of 2015-2016 per birth cohort and the *département*’s BTV-8 status

Birth cohort	BTV-8 non-infected *départements*							BTV-8 infected *départements*					
	No. analyses	ELISA positive			ELISA doubtful			No. analyses	ELISA positive			ELISA doubtful	
		No.	% (CI 95%)		No.	% (CI 95%)			No.	% (CI 95%)		No.	% (CI 95%)
< July 2008	1310	1254	95.7 [94.5 - 96.8]		4	0.3 [0.1 - 0.8]		866	814	94.0 [92.2 - 95.5]		2	0.2 [0 - 0.8]
July 2008 - June 2009	467	368	78.8 [74.8 - 82.4]		16	3.4 [2.0 - 5.5]		226	163	72.1 [65.8 - 77.9]		9	4.0 [1.8 - 7.4]
July 2009 - June 2010	617	194	31.4 [27.8 - 35.3]		23	3.7 [2.4 - 5.5]		317	108	34.1 [28.9 - 39.6]		6	1.9 [0.7 - 4.1]
July 2010 - June 2011	718	76	10.6 [8.4 - 13.1]		7	1.0 [0.4 - 2.0]		438	72	16.4 [13.1 - 20.2]		10	2.3 [1.1 - 4.1]
July 2011 - June 2012	1134	37	3.3 [2.3 - 4.5]		5	0.4 [0.1 - 1.0]		710	57	8.0 [6.1 - 10.3]		13	1.8 [1.0 - 3.1]
July 2012 - June 2013	4342	37	0.9 [0.6 - 1.2]		15	0.3 [0.2 - 0.6]		3179	75	2.3 [1.9 - 2.9]		2	0.1 [0 - 0.2]
> June 2013*	5428	25	0.5 [0.4 - 0.7]		25	0.5 [0.3 - 0.7]		3891	102	2.6 [2.1 - 3.2]		5	0.1 [0 - 0.3]

*Cattle > 12 months old

Seroprevalence was high (> 75%) among cattle that were present during mandatory vaccination campaigns in 2008-2010 and the period of intense BTV circulation in 2007-2009. It was lower for cattle born between July 2008 and July 2009 (76.6%, 95% CI [73.3 – 79.7]) than those born before July 2008 (95.0%, 95% CI [94.0 – 95.9]) (Chi^2^ = 11, df = 1, *p* < 0.001). Seroprevalence in animals born before 2008 did not vary greatly between regions (difference of 13%) with a minimum of 85.6% (Table 4). Conversely, a higher regional variability (difference of 25%) was observed for seroprevalence in cattle born between July 2008 and July 2009 which varied between 65.7% and 90.4%, but with a lower precision (median of the 95% CI range of 20.9%, Table 4).

**Table 4:** BTV-8 seroprevalence in cattle in the winter of 2015-2016 per birth cohort and region in France

Birth cohort	Proportion of positive results per region (%)				Proportion of doubtful results per region (%)			
	Median	Min - Max	Range	Median of the 95% CI range	Median	Min - Max	Range	Median of the 95% CI range
< July 2008	96.5	85.6 - 99.1	13.5	5.7	0.0	0.0 – 2.3	2.3	2.1
July 2008 - June 2009	75.4	65.7 – 90.4	24.7	20.9	3.6	1.2 – 5.4	4.2	10.5
July 2009 - June 2010	30.1	19.5 – 53.7	34.2	19.3	3.0	0.0 – 8.3	8.3	8.5
July 2010 - June 2011	11.9	1.1 – 21.7	20.6	13.4	1.6	0.0 – 3.6	3.6	4.5
July 2011 - June 2012	5.8	0.0 – 9.6	9.6	7.2	1.0	0.0 – 1.8	1.8	4.0
July 2012 - June 2013	1.0	0.2 – 4.6	2.6	1.7	0.0	0.0 - 1.4	1.4	0.6
> June 2013*	0.7	0.0 – 4.6	4.6	1.2	0.2	0.0 - 1.4	1.4	0.6

*Cattle > 12 months old

As expected, seroprevalence was lower (< 40%) for cattle born in 2009-2010 and 2010-2011 (Table 3) given the lower proportion of cattle vaccinated during the two voluntary vaccination campaigns in 2010-2012 (20-30% in 2010-2011). These animals were only present during voluntary vaccination campaigns or were less than 12 months old during the previous mandatory vaccination campaigns and therefore not necessarily vaccinated at that time. Animals born in 2009-2010 had a seroprevalence of 32.3% (95% CI [29.3 – 35.4]) varying between 19.5 and 53.7% according to the region, whereas those born in 2010-2011 had a seroprevalence of 12.8% (95% CI [10.9 – 14.9]) varying between 1.1 and 21.7% according to the region (Tables 3 and 4).

The proportion of doubtful ELISA results varied between 0.2 and 3.6% according to birth period (Table 3). A higher proportion and a higher regional variability of doubtful results were observed for animals born between July 2008 and June 2010 (3.3% of doubtful results, which is significantly different from other classes Chi^2^ = 233, df = 1, *p*-value< 0.001; 4-8% of regional variability; Tables 3 and 4) and those born between July 2010 and June 2012 (1.1% of doubtful results, which is significantly different from other classes Chi^2^ = 37, df = 1, p-value< 0.001; 2% of regional variability; Tables 3 and 4). This proportion was higher in infected *départements* in 2015 than in non-infected ones for cattle born between July 2010 and June 2012 (2.5% vs. 0.6%, Chi^2^ = 15.6, df = 1, p-value< 0.001, Table 3).

### Estimates of seroprevalence in the French cattle population in 2015 at national, regional and *département* levels

In July 2015, 59% of cattle in mainland France were born after June 2012, including 29% of calves less than 12 months old. The proportion of animals born before July 2008, in 2008-2009, 2009-2010, 2010-2011 and 2011-2012 was respectively 12%, 5%, 6%, 8% and 10%. The structure of the cattle population varied per *département* but this was mainly due to the proportion of animals born before July 2008 (from 6.3% to 27%) and after June 2012 (from 44% to 67%). For other birth cohorts, smaller variations were reported (less than 5%).

**Fig. 2:**
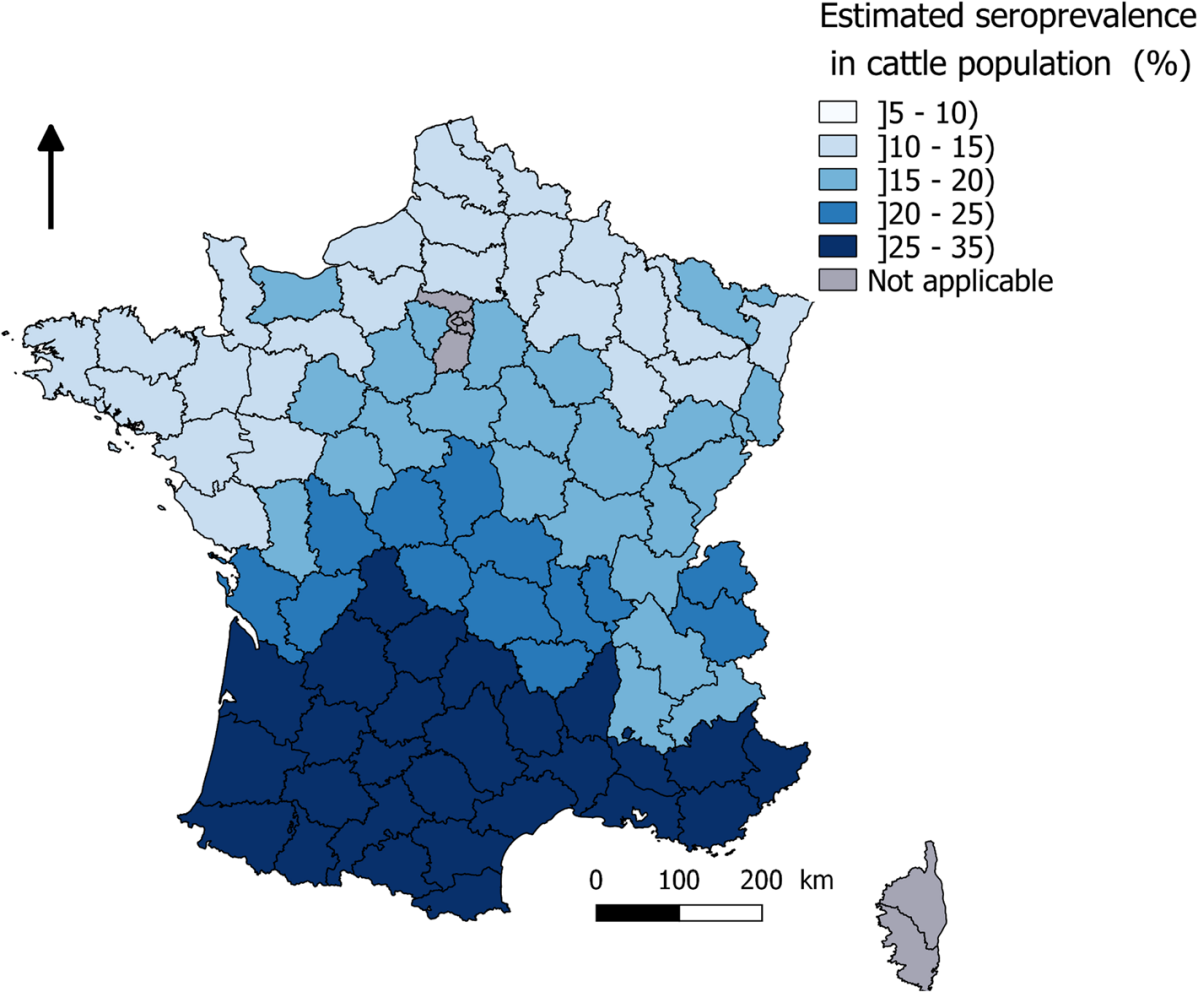
Estimation of BTV seroprevalence in cattle per French *département* in July 2015

We estimated a BT seroprevalence for the cattle population of 18.5% [CI 17.6 - 19.4] and 19.6% [CI 18.2 - 21.1] respectively in “non-infected” and “infected” *départements* in 2015. When using seroprevalence levels observed for each birth cohort per region, the proportion of seropositive animals per region in the cattle population of 2015 was estimated to range from 12.3% [10.8% – 14.6%] to 25.2% [22.3% – 28.1%] with a median of 15.7%. A higher seroprevalence (> 20%) was observed in the Auvergne and Aquitaine regions, where there was a higher proportion of beef farms and a higher proportion of older animals (> 20% of animals born before 2008). When using national seroprevalence figures per birth cohort, the seroprevalence per *département* was estimated to vary from 12.4% [11.6% – 13.2%] to 32.3% [31.3% – 33.2%] with a median of 18.1% at *département* level (Fig. 2). There was an increasing North-South gradient in the proportion of seropositive cattle per *département*. In the Allier and Puy-de-Dôme *départements* where BT outbreaks were mainly detected in 2015-2016, the seroprevalence was estimated to be 20.1 and 23.3% respectively.

### Proportion of infected cattle per birth period on infected farms in autumn 2015

On infected farms in 2015, RT-PCR prevalence was the highest in animals born between July 2012 and June 2013 (19%) and decreased in older birth cohorts down to 0% for animals born before 2008 (Table 5).

**Table 5:** RT-PCR results for cattle per birth cohort on farms found to be infected from September to October 2015 in France

Birth period	No. analyses	No. positives	Proportion of positive results (% [95 CI])
< July 2008	201	0	0 [0 - 1.8]
July 2008 - June 2009	66	3	4.5 [0.9 - 12.7]
July 2009 - June 2010	89	6	6.7 [2.5 - 14.1]
July 2010 - June 2011	113	10	8.8 [4.3 - 15.7]
July 2011 - June 2012	177	24	13.5 [8.9 - 19.5]
July 2012 - June 2013	206	39	18.9 [13.8 – 25.0]
July 2013-September 2014	180	24	13.3 [8.7 – 19.2]

## Discussion

Despite two massive mandatory vaccination campaigns in 2008-2010 where more than 90% of the cattle population was vaccinated, BTV-8 was again detected in France in 2015. We found an effect of birth cohorts on the seroprevalence related to their difference in exposure levels to BTV-8 or vaccination.

Our results suggest that BTV-8 circulation in 2015 was low and had little influence on the seroprevalence observed in cattle in the winter of 2015-2016. Seroprevalence in animals born after the vaccination ban in June 2013 can be used as an indicator of the level of recent viral circulation after this date. In *départements* where BTV circulation was evidenced in 2015-2016, 3% of those animals were seropositive. We observed similar seroprevalence levels for animals born between July 2012 and June 2013, suggesting poor vaccination coverage in 2012-2013. The difference in seroprevalence observed in the two birth cohorts of July 2010 to June 2012 between infected and non-infected *départements* (around 5%) was similar to the seroprevalence observed in young animals born after June 2012 in infected *départements* (around 3%). In 2015 or earlier, the virus may have been re-circulating in infected areas at a very low level with a maximum cumulative proportion of infected animals of 5% in all *départements* infected in 2015-2016. It is worth noting that seroprevalence in young animals born after 2013 was higher (5-10%) in only two *départements* (Allier and Puy-de-Dôme in central France) considered as the epicentre of the 2015-2016 resurgence (data not shown). A very low BTV circulation in winter 2014-2015 (< 5% of infected animals among those born after the 2013 vaccination ban) has also been evidenced by a recent study conducted in seven *départements* including the Allier [8]. Therefore, although some *départements* in our study might be wrongly classified as non-infected due to undetected current or past BTV circulation, our study shows that viral circulation in 2015 did not significantly increase the proportion of seropositive animals. In these *départements*, the presence of BT antibodies is more likely to be due to past natural infection from 2007 to 2009 and/or vaccination from 2007 to 2012 and thus existed before the 2015 resurgence.

Seroprevalence was higher (more than 75%) for animals that were present during periods of intensive viral circulation from 2007 to 2009 or mandatory vaccination campaigns when 90% of cattle were vaccinated (i.e. animals born before July 2009) and lower for those only present during voluntary campaigns. This is consistent with previous reports of the long persistence of BTV-8 antibodies after natural infection or vaccination [18, 19, 21]. Our results suggest that high vaccination coverage in 2008-2010 allowed the seroprevalence level over mainland France to be homogenised, and most animals born before July 2008 and vaccinated during two consecutive years (probably > 90%) still carried antibodies six years later. Indeed, we found little variation in seroprevalence between regions (10–25%), similar to the regional variation estimated for vaccination coverage in 2009-2010 (20%). This spatial variation is lower than could be expected if the persistence of antibodies arises only from natural infection, given the proportion of notified outbreaks with a strong spatial variation in 2007-2009 (between 3.5 to 100% per region (MAAF unpublished data)).

Being younger than 12 months during the period of vaccination or the 2007/2009 epidemics appeared to influence 2015-2016 seroprevalence levels. Only 32% of animals born between July 2009 and June 2010 and thus being less than 12 months old during the mandatory vaccination campaign of 2009-2010 were seropositive, whereas a large majority was considered to have been vaccinated in 2009-2010 (> 90%). This proportion is closer to the estimate of vaccination coverage in 2010-2011 (around 30%). This “age effect” (i.e. cattle being younger than 12 months during the period of vaccination or intense BTV circulation) can be explained by a lower exposure to viral circulation and vaccination or by the absence of a long-term immune response after vaccination or natural infection for some calves born to immune dams (either infected or vaccinated) [34, 36]. Due to the persistence of colostral antibodies that interfered with the induction of the immune response after vaccination, Vitour et al. (2011) [34] predicted a vaccination success rate of only 50% for calves of 5-6 months old born to immune dams. As the requirement was to vaccinate all calves before they reached 6 months of age and most of them were probably not re-vaccinated later during the voluntary campaigns, it is likely that some of them did not develop an effective immune response. Similarly, among animals present during the two mandatory vaccination campaigns, animals born within the year of the first campaign from July 2008 to June 2009 presented a lower seroprevalence (77%) than those aged more than 12 months (95%). In addition to the “age effect” during the vaccination period, we also attributed this difference to a faster decline in antibodies among animals vaccinated only once (prime and boost injection against serotypes 1 and 8) compared to those vaccinated twice. The higher proportion of doubtful results for animals born in 2008-2009 and 2009-2010 compared to those born before 2008 might also suggest a lower serological response and a shorter persistence of antibodies in animals vaccinated only once. Although this decline might be slightly faster, it is worth pointing out that a high proportion of those animals still had antibodies five years later.

We used group-specific cELISA to infer the immune status of cattle towards BT although the level of antibodies detected is not type-specific and not directly correlated to protection. Given that vaccination was directed against both serotypes BTV-8 and BTV-1 throughout mainland France, ELISA-positive animals can have antibodies against either or both serotypes. On infected farms in autumn 2015, we found that older birth cohorts were infected less frequently than younger ones. As discussed above, we found in parallel that older birth cohorts were also those having the highest proportion of animals with pre-existing BT antibodies. None of the animals born before 2008 for which we observed in parallel high seroprevalence levels (> 95%) were found to be infected, for example. Conversely, 19% of naive animals born in 2012-2013 were infected in autumn 2015. These results suggest that most animals with pre-existing group-specific BT antibodies detected by ELISA were protected against BTV-8 infection in 2015. Moreover, the ID-VET ELISA kits used for 96% of the study’s serological analyses offered both high sensitivity and specificity (> 99.8%) [37]. Therefore, we considered within the framework of this study that the undetected proportion of seropositive animals was probably low.

Given the respective proportion of animals per birth cohort in the cattle population, we estimated that 18-20% (resp. from 12 to 25%) of cattle were seropositive and hence probably immune in 2015 at the national level (resp. at the regional level). Our results indicated that the regional variation in cattle seroprevalence in 2015 was due more to regional variations in the cattle’s age-population structure than in seroprevalence per birth cohort. These regional seroprevalence variations are mostly explained by the spatial variation in the proportion of animals born before 2008 (from 6.7 to 18.3% per region), highly seropositive, and in the proportion of naive young animals born after 2012 (from 56.7 to 64.5% per region). In considering only the spatial variation of the cattle population structure, we estimated that from 12 to 32% of the animals were approximately still seropositive and probably immune to BTV-8 in 2015 at *département* level before the resurgence. In central France, and more specifically in the Allier and Puy-de-Dôme *départements,* where most BT outbreaks were detected in 2015-2016, this proportion was estimated to be around 20% to 25%. Estimates of the proportion of immune cattle followed an increasing North-South gradient related to variations in cattle population structure and livestock farming types: most farms located in central and southern France are beef cattle farms with a lower turn-over (older and therefore immune cattle) compared to dairy cattle farms located in northern and western France. In our estimations, we considered all calves as naive whereas some of them — born to seropositive dams — may have colostral antibodies and hence be immune from re-infection. This proportion was unknown, but by considering that only calves less than 7 months old and born to dams born before 2009 might have colostral antibodies to BT, we might have underestimated the overall seroprevalence in the cattle population by not more than 10% (BDNI data).

## Conclusion

Our results indicate that at the time of t BTV-8 resurgence in 2015 in central France, the viral circulation in naive animal birth cohorts was limited (seroprevalence < 5-10%) and from 12% to 32% of the cattle population was probably immune. The long persistence of BT antibodies after natural infection or vaccination seems to have maintained a large proportion of immune animals in the cattle population which were protected from re-infection for many years. In 2015, the proportion of immune cattle seemed to be sufficiently high to limit BTV-8 circulation. There is no reliable information on the French sheep population to accurately estimate the levels of the residual sheep herd immunity in 2015. Considering their lower number in France and lower attractiveness to Culicoides, it has probably contributed less to slowing down BTV circulation than cattle herd immunity. However, further studies with dynamic mathematical modelling, for instance, might help to solve this issue. The immunity of the cattle population would continue to decrease along with the population turnover in non-infected areas. Based on our results and the respective proportion of each birth cohort in the cattle population of 2016-2017, we can hypothesise that 6% to 9% of the cattle population was still immune in those areas in 2016-2017. In the summer and autumn of 2016, the lower herd immunity and the higher number of infection sources may partly explain the higher BTV circulation observed in 2016 than in 2015 (1200 outbreaks between July and December 2016 vs. 284 outbreaks between August 2015 and June 2016) [29].

## Additional file

Additional file 1: Sampling design of the serological survey in winter 2015-2016 for cattle born after January 2013. (DOCX 18 kb)

## Abbreviations

BDNI: National Identification Database
BT: Bluetongue
BTV: Bluetongue virus
BTV-1: Bluetongue virus serotype 1
BTV-8: Bluetongue virus serotype 8
CI: Confidence interval
ELISA: enzyme-linked immunosorbent assay
RT-PCR: Real time polymerase chain reaction
