# Supplementary material for: Estimation of French cattle herd immunity against bluetongue serotype 8 at the time of its re-emergence in 2015
Source: BMC Vet Res. 2018 Mar 2;14:65. doi: 10.1186/s12917-018-1388-1 (PMC5834897; doi:10.1186/s12917-018-1388-1)
Supplement: Supplementary file 1 — Sampling design of the serological survey in winter 2015-2016 for cattle born after January 2013. (DOCX 18 kb) [file 12917_2018_1388_MOESM1_ESM.docx]

**Additionnal file 1. Sampling design of the serological survey in winter 2015-2016 for cattle born after January 2013.**

A national serological survey was organised by the French Ministry of Agriculture and carried out from December 2015 to March 2016 (technical instruction DGAL/SDSPA/2016-35). The objectives of this survey were to detect the presence of BTV in non-infected *départements* and to demonstrate *seasonally-free zones* of BT within the restriction zone (i.e. absence of virus circulation during the vector inactivity period). Animals born after January 2013 were targeted as they were considered to be naive (not previously exposed) and not vaccinated against BT; calves of less than 12 months old were excluded due to the potential persistence of colostral antibodies [1]. The geographical unit was the *arrondissement*, an administrative unit smaller than the *département*, with an average area of 1,600 km².

In non-infected *arrondissements*, a sampling scheme was designed to allow for the detection of a minimum animal prevalence of 5% in considering a 5% alpha error and a test sensitivity of 99.8%, according to the formula of Cannon, 2001 [2]. Accordingly, sixty animals were sampled per *arrondissement*.

In infected *arrondissements*, a sampling scheme was designed to allow for the detection of a minimum monthly animal incidence of 5% in considering a 5% alpha error and a test sensitivity of 99.8%, according to the formula of Cannon, 2001. The objective was to monitor during two consecutive months 60 animals which were found seronegative during the first sampling. In order to have 60 seronegative animals to be monitored, the sample size of the first sampling were calculated in considering a maximum herd seroprevalence of 50% in the most infected *arrondissements* (*arrondissement* with ≥10 outbreaks), a herd seroprevalence of 10% in other infected *arrondissements* and a maximum of 15 animals that could be lost between the two successive samplings. Sample size per *arrondissement* was respectively 150 and 105 cattle for most infected *arrondissements* and other infected *arrondissements.*

**References**

1. Vitour D, Guillotin J, Sailleau C, Viarouge C, Desprat A, Wolff F, et al. Colostral antibody induced interference of inactivated bluetongue serotype-8 vaccines in calves. Vet Res. 2011;42:18.

2. Cannon RM. Sense and sensitivity — designing surveys based on an imperfect test. Prev Vet Med. 2001;49:141–63.
